# The Effects of Differentially-Expressed Homeobox Family Genes on the Prognosis and HOXC6 on Immune Microenvironment Orchestration in Colorectal Cancer

**DOI:** 10.3389/fimmu.2021.781221

**Published:** 2021-12-07

**Authors:** Lina Qi, Chenyang Ye, Ding Zhang, Rui Bai, Shu Zheng, Wangxiong Hu, Ying Yuan

**Affiliations:** ^1^ Department of Medical Oncology, Key Laboratory of Cancer Prevention and Intervention, Ministry of Education, The Second Affiliated Hospital, Zhejiang University School of Medicine, Hangzhou, China; ^2^ Cancer Institute, Key Laboratory of Cancer Prevention and Intervention, Ministry of Education, The Second Affiliated Hospital, Zhejiang University School of Medicine, Hangzhou, China; ^3^ Cancer Center, Zhejiang University, Hangzhou, China

**Keywords:** colorectal cancer, homeobox gene family, prognosis, HOXC6, tumor microenvironment

## Abstract

**Background:**

The homeobox (HOX) gene family encodes highly conserved transcription factors, that play important roles in the morphogenesis and embryonic development of vertebrates. Mammals have four similar HOX gene clusters, HOXA, HOXB, HOXC, and HOXD, which are located on chromosomes 7, 17,12 and 2 and consist of 38 genes. Some of these genes were found to be significantly related to a variety of tumors; however, it remains unknown whether abnormal expression of the HOX gene family affects prognosis and the tumor microenvironment (TME) reshaping in colorectal cancer (CRC). Therefore, we conducted this systematic exploration to provide additional information for the above questions.

**Methods:**

RNA sequencing data from The Cancer Genome Atlas (TCGA) and mRNA expression data from Gene Expression Omnibus (GEO) combined with online tumor analysis databases (UALCAN, TIMER, PrognoScan) were utilized to explore the relationship among abnormal expression of HOX family genes, prognosis and the tumor immune microenvironment in CRC.

**Results:**

1. Differential expression and prognosis analysis: 24 genes were significantly differentially expressed in CRC compared to adjacent normal tissues, and seven upregulated genes were significantly associated with poor survival. Among these seven genes, univariate and multivariate Cox regression analysis revealed that only high expression of HOXC6 significantly contributed to poor prognosis; 2. The influence of overexpressed HOXC6 on the pathway and TME: High HOXC6 expression was significantly related to the cytokine pathway and expression of T cell attraction chemokines, the infiltration ratio of immune cells, expression of immune checkpoint markers, tumor mutation burden (TMB) scores and microsatellite instability-high (MSI-H) scores; 3. Stratified analysis based on stages: In stage IV, HOXC6 overexpression had no significant impact on TMB, MSI-H, infiltration ratio of immune cells and response prediction of immune checkpoint blockers (ICBs), which contributed to significantly poor overall survival (OS).

**Conclusion:**

Seven differentially expressed HOX family genes had significantly worse prognoses. Among them, overexpressed HOXC6 contributed the most to poor OS. High expression of HOXC6 was significantly associated with high immunogenicity in nonmetastatic CRC. Further research on HOXC6 is therefore worthwhile to provide potential alternatives in CRC immunotherapy.

## Introduction

As one of the most common digestive system malignant tumors, the incidence and mortality of CRC ranks third among various solid cancers worldwide (1). The main cause of death is metastasis. According to statistics, 20% of patients are diagnosed with advanced stage cancer and 25-30% of patients with stage I/II cancer suffer relapse within 5 years after a curative operation (2). In addition to traditional therapies, a variety of new methods, such as immunotherapy and targeted therapy have shown breakthrough effects. In 2015, Le and his colleagues found that pembrolizumab can bring exciting clinical benefits to dMMR/MSI-H metastatic CRC (mCRC), but its objective response rate (ORR) remained only 40% (3). Except for dMMR/MSI-H, new predictive biomarkers of immunotherapy efficacy in colorectal cancer are on the way, such as elevated TMB, POLE/POLD1 mutations, and ARID1A mutation (4–6). However, these current biomarkers are far from meeting the needs of patient screening who may benefit from immunotherapy. Further studies on the tumor microenvironment may provide clues for revealing the cause of tumor immune escape and developing new immunotherapy targets.

The HOX gene family encodes highly conserved transcription factors, that play important roles in the morphogenesis and embryonic development of vertebrates. Mammals have four similar homeobox gene clusters (HOXA, HOXB, HOXC, and HOXD), which consist of 38 genes and are located on chromosomes 7, 17,12 and 2. Many studies have revealed that diverse HOX genes can either inhibit or promote the development of tumors on the basis of their abnormal expression in certain organs: HOXB family in breast cancer (7), HOXA13 in gastric cancers (8), HOXB5 in leukemia (9) and hepatocellular carcinoma (10). In CRC, very few studies have focused on HOX genes. HOXA13, HOXD13 and HOXC6 were reported to promote cancer progression in CRC (11–13), and HOXB13 was reported to suppress tumors in CRC (14).

However, systematic studies on HOX gene family in CRC remain unclear. Therefore, we conducted this systematic analysis to explore whether the 38 HOX family genes were differentially expressed in CRC. Moreover, we further evaluated their prognostic values and TME orchestration abilities.

## Material and Methods

### Data Downloaded From TCGA

All level3 CRC RNA-Seq data and corresponding clinical information were obtained from TCGA dataset, in which the method of acquisition and application complied with the guidelines and policies. mRNA-seq data were analyzed in TPM format converted from counts.

### Differential Expression Analysis of HOX Family Genes Between CRC and Normal Colon Tissues

UALCAN (www.ualcan.path.uab.edu), an online TCGA analysis database, was used for differential expression analysis of HOX family genes in CRC samples.),

GEO database: Expression microarray datasets GSE21815 and GSE37182 were used for differential expression analysis between CRC and normal colon tissues. 9 normal colon tissues and 132 CRC tissues were enrolled in GSE21815 (Platform: GPL6480, Agilent-014850 Whole Human Genome Microarray 4x44K G4112F). 88 normal colon tissues and 84 CRC tissues were enrolled in GSE37182 (Platform: GPL6947, Illumina HumanHT-12 V3.0 expression beadchip).

### Prognostic Analysis of Differentially-Expressed Genes

TCGA database: For Kaplan-Meier curves, p-values and hazard ratio (HR) with 95% confidence interval (CI) were generated by log-rank tests and univariate Cox proportional hazards regression. The KM survival analysis with log-rank test were also used to compare the survival difference between above two groups. The whole cohort was divided into two or three groups equally according to HOXC6 expression values. Univariate and multivariate cox regression analysis was performed to identify the prognostic values of genes expression.

GEO database: GSE17536 and GSE12945 were used to validate the survival difference between top 25% HOXC6 high expression and top 25% HOXC6 low expression groups. KM plotter was drawn by GraphPad Prism 8.0.

### Volcano Plots, GO, KEGG and GSEA Analysis

Limma package (version: 3.40.2) of R software was used to study the differential expression of mRNAs. The adjusted P-value was analyzed to correct for false positive results in TCGA or GTEx. “Adjusted P < 0.05 and Log (Fold Change) >1 or Log (Fold Change) < -1” were defined as the thresholds for the screening of differential expression of mRNAs between top 25% HOXC6 high expression and top 25% HOXC6 low expression groups.

Gene Ontology (GO) is a widely-used tool for annotating genes with functions, especially molecular function (MF), biological pathways (BP), and cellular components (CC). Kyoto Encyclopedia of Genes and Genomes (KEGG) Enrichment Analysis is a practical resource for analytical study of gene functions and associated high-level genome functional information. To better understand the carcinogenesis of mRNA, ClusterProfiler package (version: 3.18.0) in R was employed to analyze the GO function of potential targets and enrich the KEGG pathway.

Hallmark gene sets from the molecular signatures database (MSigDB) were used to determine whether any signatures were enriched in specific groups by gene set enrichment analysis (GSEA). Significantly enriched hallmarks were chosen according to a *P*-value < 0.05.

### Effect of HOXC6 Expression on the Characteristics of Tumor Immune Microenvironment

TIMER (Tumor IMmune Estimation Resource, https://cistrome.shinyapps.io/timer), a web server for comprehensive analysis of tumor-infiltrating immune cells, was used to analyze the correlation of HOXC6 expression and chemokine expression, immune cell infiltration ratio, immune checkpoint marker expression. TIMER2.0 was used to analyze the immune cell infiltration ratio calculated by 5 different algorithms.

TCGA database: Immunedeconv, an R package including CIBERSORT algorithm was utilized to make reliable immune infiltration estimations. PDL1, CTLA4, TIM3, LAG3, PD1, PDL2 and TIGIT were selected as immune-checkpoint-relevant transcripts. CCL2, CCL3, CCL4, CCL5, CXCL9, CXCL10 and CXCL11 were selected as T cell attractive chemokines (15). The expression values of these 14 genes were extracted. Spearman’s correlation analysis was used to analyze the correlation between HOXC6 expression and TMB/MSI scores (16). The whole cohort was divided into two groups equally according to HOXC6 expression values.

SNP analysis: Somatic variants in CRC was analyzed by Maftools, which is an efficient and comprehensive tool for analysis of somatic variants in cancer (17). The whole cohort was divided into two groups equally according to HOXC6 expression values. However, data was missing from several samples which resulted in unequal number in HOXC6 high (N=232) and low (N=167) groups.

### Interaction Between CD8+ T and RKO Cells

Cell culture: The RKO cell line was purchased from ATCC and cultured with RPMI 1640 medium (Gibco) containing 10% fetal bovine serum (FBS, BI Industry). The cells were incubated at 37°C with 5% CO2.

Stable gene overexpression was constructed by lentiviral transfection system: HOXC6-overexpressed and negative control lentivirus were purchased from GeneChem (Shanghai, China). For infection, 10^5^ cells were plated into 6-well plates and cocultured with 2.5 x 10^6^ transducing-units (TU) virus in the presence of 1X HitransG (GeneChem, Shanghai, China) and standard medium. Twelve to 15 hours later, the medium was replaced with fresh complete culture medium. After 72h of transfection, 2mg/ml puromycin was added to the culture medium for RKO selection. Quantitative reverse transcription-polymerase chain reaction (qRT-PCR) were utilized to confirm HOXC6 overexpression.

CD8+ T induction from Peripheral Blood Mononuclear Cells (PBMC): PBMCs were isolated from a healthy donor’s peripheral blood using Ficoll (GE Healthcare) following a standard protocol. PBMCs were cultured with RPMI 1640 medium (Gibco) containing 10% fetal bovine serum (FBS, BI Industry) inactivated by 56°C water bath, PHA (1 ug/ml), and IL-2 (10 ng/ml) for 3 days. Then the culture medium was replaced by RPMI 1640 medium (Gibco) containing 10% fetal bovine serum (FBS, BI Industry) inactivated by 56°C water bath, OKT3 (50 ng/ml), and IL-2 (10 ng/ml) for every 2 days. The cells were incubated at 37°C with 5% CO2. After 1 week inducement, cells were harvested for coculture experiment and induction identification was measured by flow cytometry and qRT- PCR.

Noncontact coculture of CD8+ T cells and RKO tumor cells: CD8+ T cells and RKO cells coculture were conducted with the noncontact coculture Transwell system (Corning, USA). Inserts containing 1.0 × 10^7^ CD8+ T cells were transferred to 6-well plates previously seeded with RKO cells (2.5 × 10^5^ cells per well) and cocultured in 1.5% FBS-containing medium for 72h. After coculture, CD8+ T and RKO cells were harvested for RNA extraction.

RNA extraction and qRT- PCR: Total RNA was extracted from RKO and CD8+ T cells using Trizol following a standard protocol. The Takara PrimeScript TM RT Master Mix Kit (Takara, RR036Q) was used for reverse transcription. The iTaq Universal SYBR Green Supermix (BioRad) and Applied Biosystems 7500 Fast Real-Time PCR System were applied for qRT- PCR. GAPDH was used as the loading control. Experiments were carried out in triplicate. The results were calculated as follows: ΔCT=CT _Experimental/NC_-CT_GAPDH_, ΔΔCT=ΔCT _Experimental/NC_-ΔCT_NC_, foldchange=2^-ΔΔCT^. The primers used for qRT-PCR are detailed in **Table S3**.

### Coexpression Analysis

The dataset used comprised HOXC6, MLH1, PMS2, MSH2 and MSH6 mRNA-seq data from TCGA tumors. Multi-gene correlation map is displayed by the R software package pheatmap. Spearman’s correlation analysis was used to describe the correlation between quantitative variables without a normal distribution. *P* < 0.05 was considered statistically significant.

### HOXC6 Expression and MLH1 Mutation Analysis

For CRC patients in TCGA database, tumor gene mutation MAF data (TCGA) was downloaded from genomic data Commons (GDC) data portal (https://portal.gdc.cancer.gov **)** (18).

### Prediction of ICB Efficacy Based on TIDE Algorithm

Potential ICB response was predicted with TIDE algorithm using raw counts of RNA-sequencing data (level 3) and corresponding clinical information of 407 nonmetastatic and 88 mCRC patients from TCGA (19). The whole cohort was divided into two groups equally according to HOXC6 expression values.

### Statistical Analysis

Known batch effects were corrected using the ComBat function in the Bioconductor sva package (20). All the above analysis methods and R package were implemented by R foundation for statistical computing (2020) version 4.0.3 and software packages ggplot2 and pheatmap. *P <*0.05 was considered statistically significant. In addition, main R software packages used in this research were detailed in supplementary methods.

## Results

### Differential Expression and Prognosis Analysis Of 38 HOX Family Genes in Colorectal Cancer

**Figure 1:**
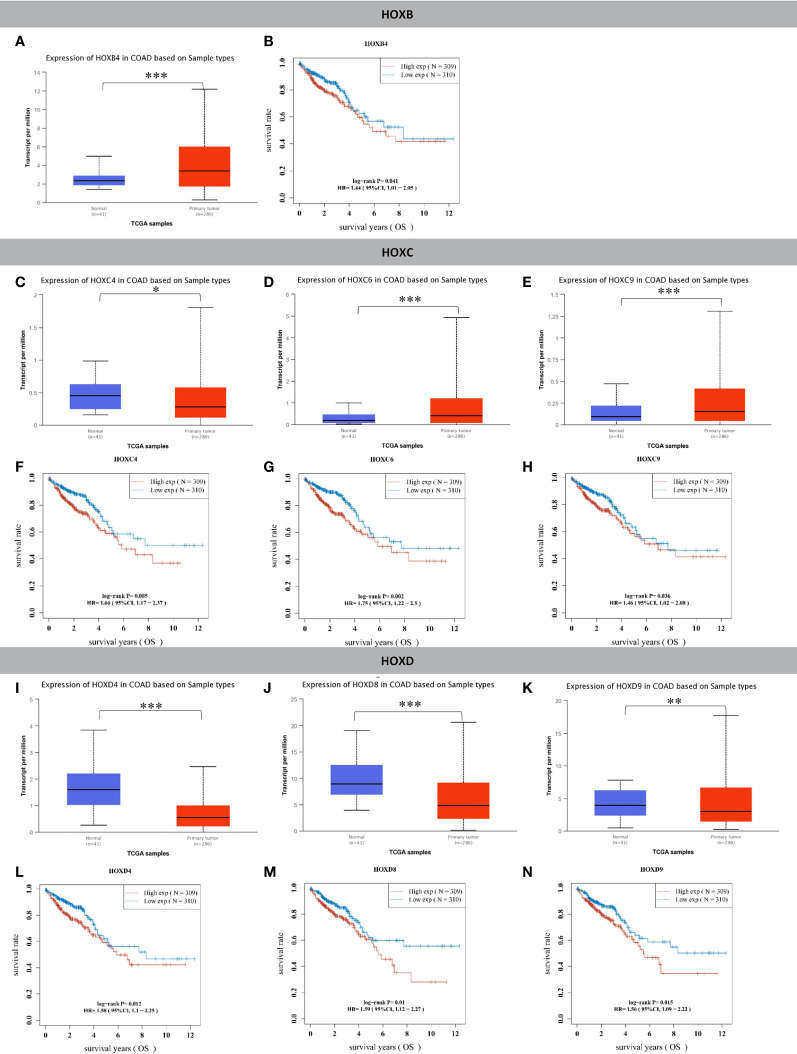
HOX family genes were significantly differentially expressed in tumor tissues compared to normal colon tissues and had significant prognostic value. **(A)** HOXB4 was significantly upregulated in colon cancer compared to normal tissues. **(B)** The HOXB4 high expression group had significantly worse OS than the low expression group in CRC. **(C)** HOXC4 was significantly downregulated in colon cancer compared to normal tissues. **(D)** HOXC6 was significantly upregulated in colon cancer compared to normal tissues. **(E)** HOXC9 was significantly upregulated in colon cancer compared to normal tissues. **(F)** The HOXC4 high expression group had significantly worse OS than the low expression group in CRC. **(G)** The HOXC6 high expression group had significantly worse OS than the low expression group in CRC. **(H)** The HOXC9 high expression group had significantly worse OS than the low expression group in CRC. **(I)** HOXD4 was significantly downregulated in colon cancer compared to normal tissues. **(J)** HOXD8 was significantly downregulated in colon cancer compared to normal tissues. **(K)** HOXD9 was significantly downregulated in colon cancer compared to normal tissues. **(L)** The HOXD4 high expression group had significantly worse OS than the low expression group in CRC. **(M)** The HOXD8 high expression group had significantly worse OS than the low expression group in CRC. **(N)** The HOXD9 high expression group had significantly worse OS than the low expression group in CRC. *P < 0.05, **P < 0.01, ***P < 0.001.

First, we performed differential expression analysis of 38 genes in the HOX gene family, and the results showed that compared with normal colon tissues, there were 15 genes whose expression was significantly upregulated (HOXA3, HOXA9, HOXA10, HOXA11, HOXB3, HOXB4, HOXB5, HOXB6, HOXB7, HOXB9, HOXC5, HOXC6, HOXC9, HOXC10, HOXC11) (**Figures 1A, D, E** and **S1**) and nine genes whose expression was significantly downregulated in tumor tissues (HOXA5, HOXA6, HOXA13, HOXC4, HOXD1, HOXD3, HOXD4, HOXD8, HOXD9) (**Figures 1C, I–K**, and **S2**). Second, we conducted a prognostic analysis of these 24 genes with significant differential expression, and the results showed that the differential expression of seven genes contributed significantly to survival (**Figures 1B, F–H, L–N**). In the analysis of these seven genes, the 4 genes HOXC4, HOXD4, HOXD8, and HOXD9 were significantly expressed at low levels in CRC, but the low expression group had a significantly better prognosis. The remaining three genes, HOXB4, HOXC6, and HOXC9 were significantly overexpressed in CRC, and the high expression groups had a significantly worse prognosis.

**Figure 2:**
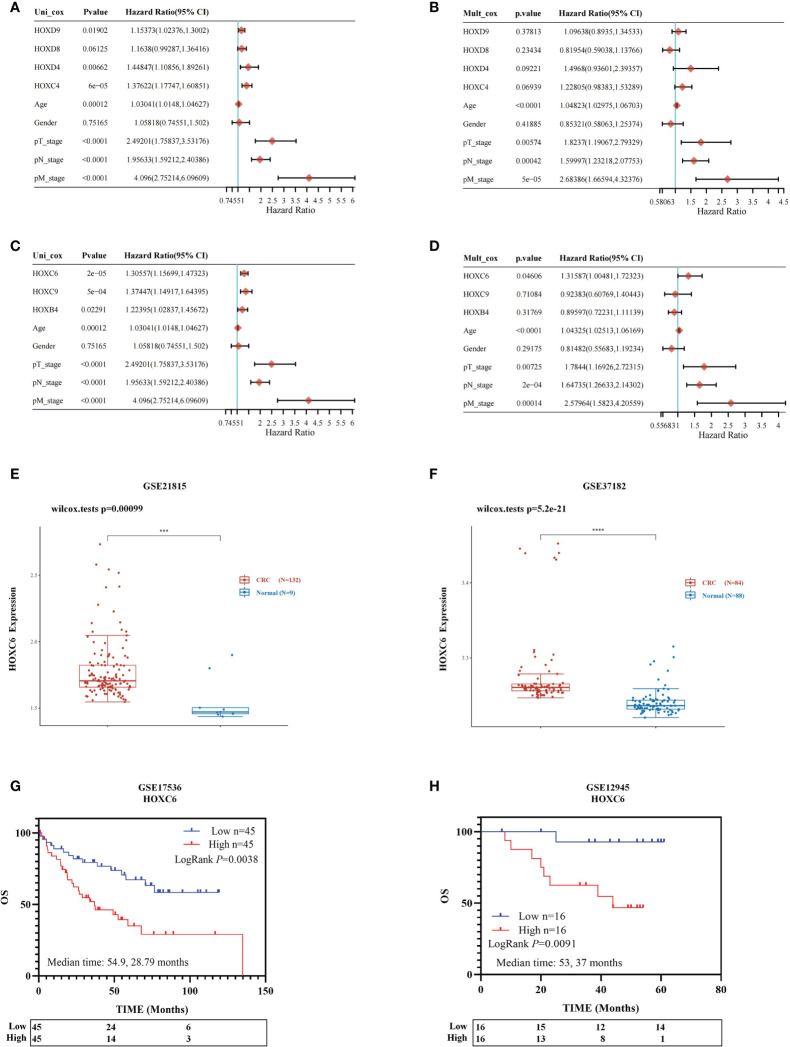
Prognostic analysis of three significantly differentially expressed genes and identification of HOXC6 upregulation and prognostic value. Univariable and multivariable Cox regression analysis for the significantly downregulated genes **(A, B)**. **(A)** Univariable Cox regression analysis revealed that upregulation of HOXC4, HOXD4 and HOXD9 contributed to poor OS. **(B)** Multivariable Cox regression analysis revealed no significant results for HOXC4, HOXD4, HOXD8 and HOXD9. Univariable and multivariable Cox regression analysis for the significantly upregulated genes **(C, D)**. **(C)** Univariable Cox regression analysis revealed that upregulation of HOXB4, HOXC6, and HOXC9 contributed to poor OS. **(D)** Multivariable Cox regression analysis revealed that upregulation of HOXC6 contributed most to poor OS among HOXB4 and HOXC9. **(E)** The GSE21815 dataset confirmed that HOXC6 was significantly upregulated in CRC compared to normal colon tissue. **(F)** The GSE37182 dataset confirmed that HOXC6 was significantly upregulated in CRC compared to normal colon tissue. **(G)**. GSE17536 dataset confirmed that upregulation of HOXC6 was significantly associated with poor OS (*P*=0.0038, median follow-up time for high and low expression groups: 54.9 and 28.79 months). **(H)** The GSE12945 dataset confirmed that upregulation of HOXC6 was significantly associated with poor OS (*P*=0.0091, median follow-up time for high and low expression groups: 53 and 37 months). ***P < 0.001.

Therefore, we performed Cox univariate and multivariate prognostic analyses on the expression levels of four significantly downregulated genes (HOXC4, HOXD4, HOXD8, HOXD9) and three significantly upregulated genes (HOXB4, HOXC6, HOXC9). Univariate analysis identified that the high expression of HOXC4, HOXD4 and HOXD9 was associated with a significantly worse prognosis (**Figure 2A**). However, high expression of these four genes did not contribute to prognosis in the multivariate analysis (**Figure 2B**). For the three significantly upregulated genes, univariate analysis revealed that these three genes were associated with a significantly worse prognosis (**Figure 2C**). In the multivariate analysis, only high expression of HOXC6 had a significant contribution to the prognosis [**Figure 2D**, *P*= 0.046, HR= 1.316 (1.0048-1.723)]. Detailed clinical information and expression data of these three genes of CRC patients from TCGA database are listed in **Table S1**.

Four independent GSE datasets were used to confirm the conclusion that HOXC6 was significantly upregulated in CRC compared to normal colon tissues and that high expression of HOXC6 contributed significantly to poor survival. The GSE21815 and GSE37182 datasets showed that the expression of HOXC6 was significantly higher in CRC than in normal colon tissues (**Figures 2E, F**), which was consistent with previous results reported by other scientists (21). The GSE17536 and GSE12945 datasets showed that compared with the HOXC6 low expression group, the HOXC6 high expression group had significantly worse OS (**Figures 2G, H**).

### The Influence of Overexpressed HOXC6 on Pathways

**Figure 3:**
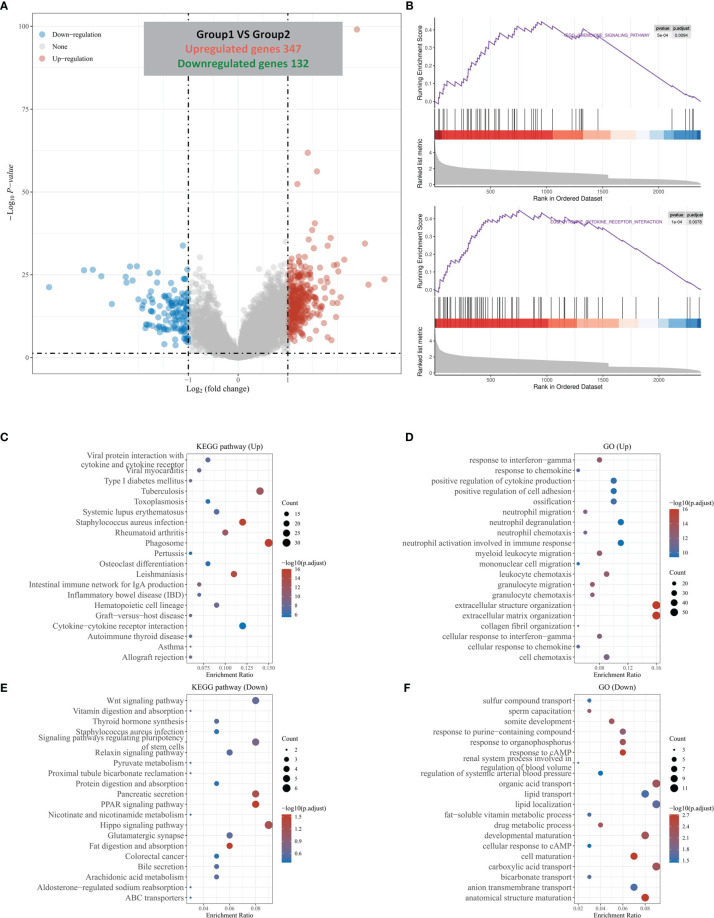
DEGs divided by HOXC6 expression and GSEA, KEGG, and GO analysis of these DEGs. **(A)** A total of 347 upregulated genes and 132 downregulated genes were identified in the HOXC6 high-expression group compared to the low-expression group. **(B)** GSEA revealed that the cytokine interaction pathway and chemokine signaling pathway were significantly enriched in DEGs by HOXC6 upregulation. **(C)** KEGG pathway analysis for 347 upregulated DEGs. **(D)** GO analysis for 347 upregulated DEGs. **(E)** KEGG pathway analysis for 132 downregulated DEGs. **(F)** GO analysis for 132 downregulated DEGs.

Through the above analysis, we found that HOXC6 was significantly overexpressed in CRC and that high expression was associated with a significantly worse prognosis. However, the changes of pathways caused by HOXC6 overexpression remain unknown. Therefore, we explored the changes in pathways. First, we analyzed the differentially expressed genes (DEGs) between the HOXC6 high and low expression groups and found that there were 347 upregulated and 132 downregulated genes in the HOXC6 high expression group compared with the HOXC6 low expression group (**Figure 3A**). To further explore the changes in pathways and biological functions caused by these DEGs, we further performed GSEA, KEGG, and GO analysis. GSEA found that HOXC6 overexpression was significantly related to chemokine signaling and cytokine receptor interactions (**Figure 3B** and **Table S2**). In KEGG analysis, we found that these upregulated genes were significantly enriched in inflammation-related pathways, such as cytokine and cytokine receptors, LPS, and IBD (**Figure 3C**). In GO analysis, these upregulated genes were mainly enriched in immune pathways, such as in response to IFN-γ, response to chemokines, and neutrophil migration (**Figure 3D**). We also performed KEGG and GO analyses on these downregulated genes, and the results are shown in **Figures 3E, F**.

In summary, overexpression of HOXC6 is likely to be associated with remodeling of the tumor immune microenvironment in CRC.

### Correlation Between HOXC6 Overexpression and TME Characteristics

The above analysis indicated that HOXC6 overexpression was likely related to orchestration of the TME. In the TME, the proportion of immune cell infiltration and functional status are significantly related to the prognosis of tumor patients and the efficacy of immune checkpoint inhibitors. Therefore, we further analyzed the correlation between HOXC6 overexpression and tumor immune microenvironment characteristics, including chemokine expression level, immune cell infiltration ratio, immune checkpoint expression level, TMB score, and MSI-H status.

**Figure 4:**
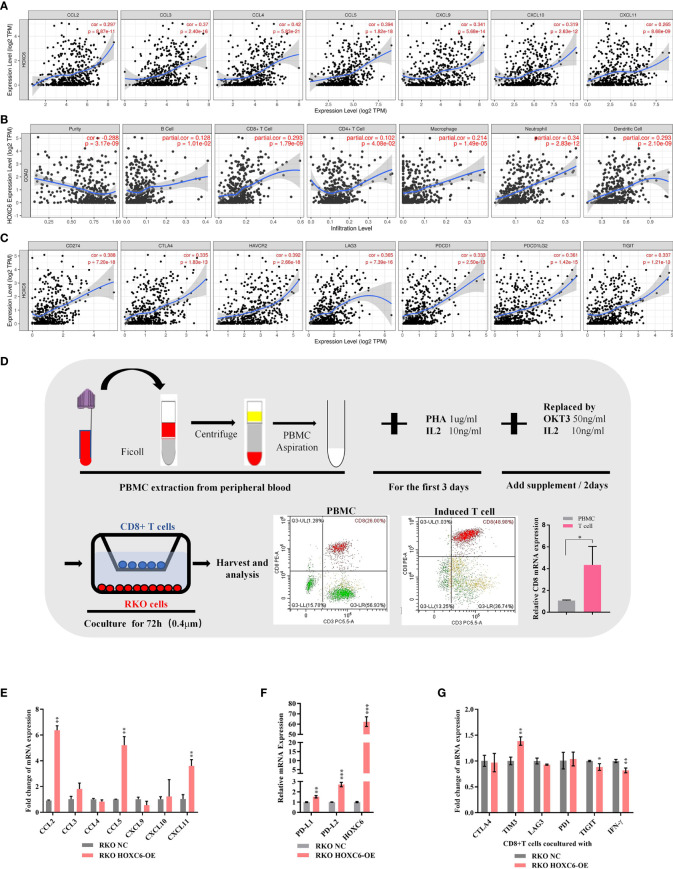
The effect of upregulated HOXC6 on the tumor immune microenvironment in CRC. **(A)** Expression of HOXC6 had a significantly positive correlation with the expression of T cell attractive chemokines in the TIMER database. **(B)** Expression of HOXC6 had a significantly positive correlation with the infiltration ratio of various immune cells in the TIMER database. **(C)** Expression of HOXC6 had a significantly positive correlation with various immune checkpoint molecules in the TIMER database. **(D)** *In vitro* induction of CD8+ T cells from PBMCs isolated from peripheral blood and identification using flow cytometry and qRT- PCR. **(E)** The expression of CCL2, CCL5, and CXCL11 was significantly upregulated in the RKO HOXC6-OE group compared to RKO NC group (both groups were cocultured with CD8+ T cells). **(F)** The expression of PD-L1 and PD-L2 was significantly upregulated in the RKO HOXC6-OE group compared to RKO NC group (both groups were cocultured with CD8+ T cells). **(G)** Expression of TIM3 was significantly upregulated and IFN-γ was significantly downregulated in CD8+ T cells cocultured with RKO HOXC6-OE group compared to RKO NC group. *P < 0.05, **P < 0.01, ***P < 0.001.

First, we used the TIMER database to analyze the correlation between the expression of HOXC6 and the expression of T cell attractive chemokines (CCL2, CCL3, CCL4, CCL5, CXCL9, CXCL10, CXCL11) (**Figure 4A**), the infiltration ratio of the main immune infiltrating cell population (B cells, CD4+T, CD8+T, macrophage, neutrophil, dendritic cell) (**Figure 4B**), and the expression levels of major immune checkpoint molecules (PDL1, CTLA4, TIM3, PD1, PDL2, TIGIT) (**Figure 4C**). The results showed that the expression of HOXC6 was significantly positively correlated with these tumor immune microenvironment characteristics (**Figures 4A–C**). To verify the above results, we performed *in vitro* coculture experiments to identify the effect of HOXC6- upregulated colon cancers on CD8+ T cells. RKO, a commonly used CRC cell line, was selected in this experiment. CD8+ T cells *in vitro* induction was performed following the protocol detailed in **Figure 4D**. Then noncontact coculture of CD8+ T cells and RKO NC/RKO HOXC6-overexpressing (HOXC6-OE) cells was performed. Overexpression of HOXC6 in RKO cells led to significant upregulation of T cell attraction chemokines (CCL2, CCL5 and CXCL11) (**Figure 4E**) and immune checkpoint moleculars (PD-L1 and PD-L2) in RKO (**Figure 4F**). Moreover, overexpression of HOXC6 in RKO cells also caused significant upregulation of TIM3 and downregulation of IFN- γ in CD8+ T cells at the mRNA level (**Figure 4G**). This means, CRC cells with high HOXC6 expression attract more CD8+ T cells by upregulating T cell attraction chemokines, however the tumor killing function of CD8+ T cells might be exhausted by downregulation of IFN- γ.

**Figure 5:**
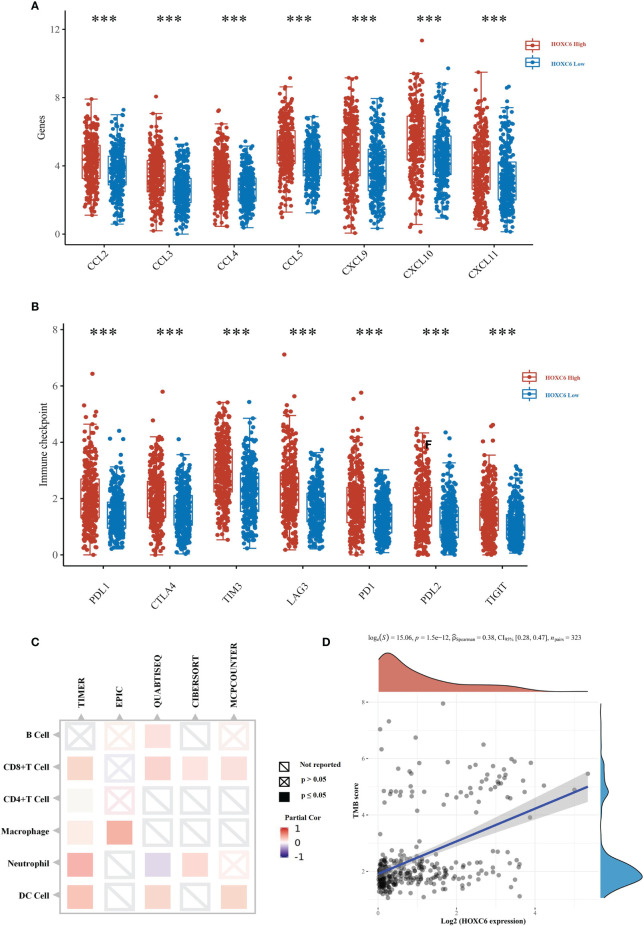
TCGA verification of the effect of upregulated HOXC6 on the tumor immune microenvironment in CRC. **(A)** The expression of T cell attractive chemokines was significantly upregulated in the HOXC6 high-expression group compared to the low-expression group. **(B)** The expression of immune checkpoint molecules was significantly upregulated in the HOXC6 high-expression group compared to the low-expression group. **(C)** Expression of HOXC6 had a significantly positive correlation with the infiltration ratio of various immune cells using different algorithms. **(D)** Expression of HOXC6 had a significantly positive correlation with TMB score (*P*<0.001, R=0.36). ***P < 0.001.

Moreover, we used the TCGA mRNA expression data and verified that the expression of immune cell attraction chemokines and immune checkpoints was significantly higher in the HOXC6 high expression group than in the low expression group (**Figures 5A, B**). To verify the proportion of immune cell infiltration, we used the TIMER2.0 database, which uses a variety of algorithms to calculate the proportion of six types immune cells. The results showed that the four algorithms all indicated that the infiltration ratio of CD8+ T cells was significantly positively correlated with high HOXC6 expression (**Figure 5C**). Studies have shown that patients with high TMB have more abundant neoantigens and higher immunogenicity in CRC (22). Accordingly, we conducted a correlation analysis on the expression of HOXC6 and TMB score, and the results showed a significant positive correlation (**Figure 5D**, *P*< 0.001, R= 0.38). What’s more, we further analyzed the tumor mutation signature between HOXC6 high and low groups and the result showed that the C> T mutation was the main type in both groups (**Figures S3A, B**). However, different from HOXC6 low expression group, proportion of T> G mutation was higher than T>A, and the Ti (Transition) mutation type was higher in HOXC6 high-expression group (**Figures S3A, B**). Percentage of mutations of specific genes, such as BRAF, RNF43, and PIK3C2B were significantly higher in HOXC6 high expression group (**Figure S3C**).

**Figure 6:**
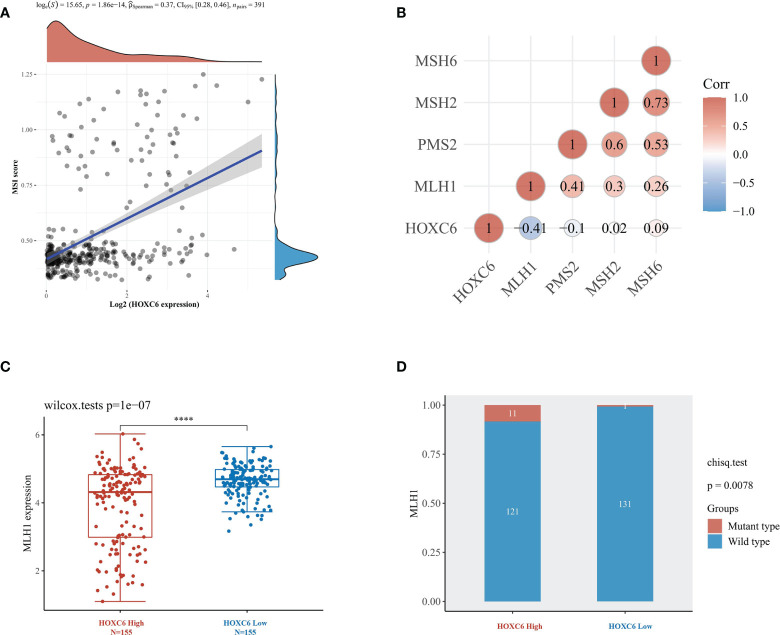
High expression of HOXC6 was significantly correlated with high MSI-H score and dMMR status in CRC. **(A)** The expression of HOXC6 had a significantly positive correlation with MSI score (*P*<0.001, R=0.36). **(B)** The expression of HOXC6 had a significantly negative correlation with the expression of MLH1 (*P*<0.001, R=-0.41). **(C)** The expression of MLH1 was significantly downregulated in the HOXC6 high-expression group compared to the low-expression group (*P*<0.001). **(D)** The number of patients with MLH1 mutations was significantly higher in the HOXC6 high-expression group than in the low-expression group (*P*=0.0078). ****P <0.0001.

In the immunotherapy of CRC, National Comprehensive Cancer Network (NCCN) and Chinese Society of Clinical Oncology (CSCO) guidelines have recommended pembrolizumab as the first-line treatment for dMMR/MSI-H mCRC patients (23). dMMR/MSI-H is an important predictor biomarker of PD1 inhibitor efficacy, so we analyzed the correlation between HOXC6 expression and dMMR/MSI-H status. First, we found that the expression of HOXC6 was significantly positively correlated with the MSI-H score (**Figure 6A**, *P*< 0.001, R= 0.37). MSI-H patients are basically dMMR, which is caused by the loss of function of the four main mismatch repair genes, of which the loss of MLH1 expression is the most common situation. Therefore, we analyzed the expression correlation of HOXC6 and four mismatch repair genes, and found that the expression of HOXC6 and MLH1 had a significant negative correlation (**Figure 6B**, *P*< 0.001, R= -0.41). In sporadic dMMR CRC, the loss of MLH1 expression caused by MLH1 promoter methylation is the main cause. However, in Lynch syndrome, the loss of MLH1 expression is caused by MLH1 mutation (24). We found that compared with the HOXC6 low expression group, the expression of MLH1 was significantly lower in the HOXC6 high expression group (**Figure 6C**, *P*< 0.001). Moreover, the number of patients with MLH1 mutations was also significantly higher in the HOXC6 high expression group (**Figure 6D**, *P*= 0.0078). Moreover, we performed *in vitro* cell line experiments and found that MLH1- knockdown caused significant upregulation of HOXC6 in microsatellite stable (MSS) CRC cell lines (HT29 and Sw620) (25). In summary, the high expression of HOXC6 had a significant correlation with dMMR/MSI-H status in CRC.

### Stratified Analysis Based on Stages

**Figure 7:**
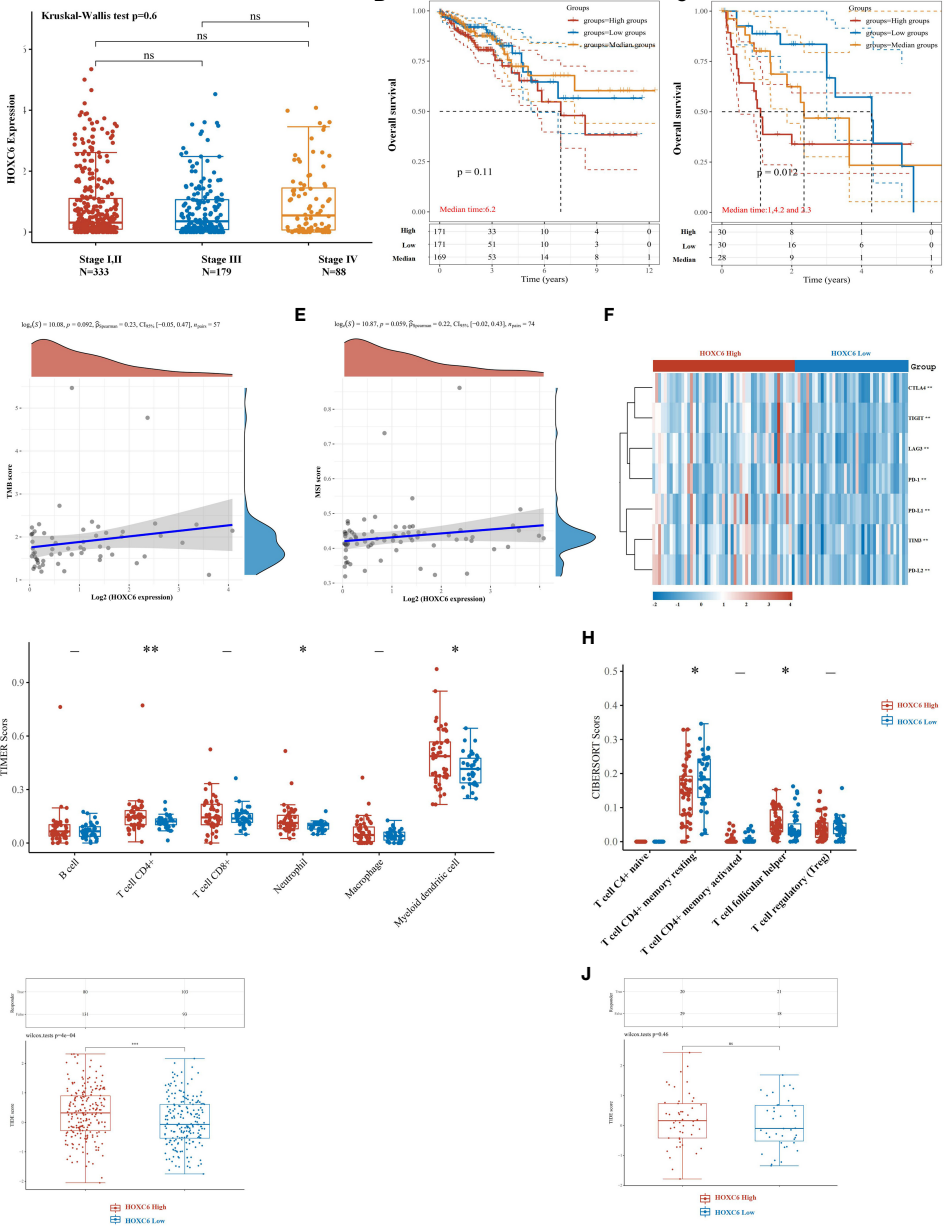
High expression of HOXC6 significantly contributed to poor survival in stage IV with weakened immune microenvironment characteristics in CRC. **(A)** There was no significant difference in the expression of HOXC6 among stage I&II, stage III, and stage IV. **(B)** In stages I, II and III, high expression of HOXC6 had no significant contribution to poor survival (*P*=0.11) (The whole cohort was divided into three groups equally according to the HOXC6 expression values. The median follow-up time for these three groups was 6.2 years). **(C)** In stage IV, high expression of HOXC6 contributed significantly to poor survival (*P*=0.012) (The whole cohort was divided into three groups equally according to the HOXC6 expression values. The median follow-up times for the high, medium and low expression groups were 1, 4.2, and 2.3 years, respectively). **(D)** Expression of HOXC6 had no significant correlation with TMB score (*P*=0.092). **(E)** Expression of HOXC6 had no significant correlation with MSI score (*P*=0.059). **(F)** Immune checkpoints related gene expression heat map, where different colors represent the expression trend. **(G)** Expression of HOXC6 had a significantly positive correlation with the infiltration ratio of CD4+ T cell, neutrophils and myeloid dendritic cells. **(H)** Expression of HOXC6 had significantly positive correlation with the infiltration ratio of T cell follicular helper (*P*<0.05). **(I)** The predicted immune response scores were significantly higher in the HOXC6-high expression group than in the HOXC6-low expression group which indicates worse predicted ICB efficacy in the HOXC6-high expression group (*P*<0.001). **(J)** The predicted immune response scores revealed no significance between the HOXC6-high expression group and HOXC6-low expression group. *P < 0.05, **P < 0.01, ns, no significance.

Furthermore, we analyzed the differential expression of HOXC6 and its prognostic value in different stages. First, we used TCGA expression data to analyze the expression of HOXC6 in stages I-IV, and the results showed that there was no significant difference in the expression of HOXC6 in different stages (**Figure 7A**). Second, in stages I, II, and III, patients were divided into three groups according to the expression level of HOXC6, followed by prognostic analysis, and the results showed that the expression level of HOXC6 had no significant prognostic impact (**Figure 7B**, *P*= 0.11). However, in stage IV, the HOXC6 high expression group had a significantly worse prognosis (**Figure 7C**, *P*= 0.012).

In stage IV patients, we analyzed the correlation between HOXC6 and TMB and MSI-H score and found that there was no significant correlation between HOXC6 and TMB and MSI-H score (**Figures 7D, E**). The expression of immune checkpoint related genes was significantly higher in HOXC6 high expression group than in the low (**Figure 7F**). In stage IV patients, compared with the HOXC6 low expression group, only CD4+ T cells, neutrophil and DC cell infiltration ratios were significantly higher in the HOXC6 high expression group (**Figure 7G**). For CD4+ T cells in several subgroups, we used CIBERSORT to evaluate the proportion of CD4+ T cells between the HOXC6 high and low groups and found that T cell follicular helper had significantly higher infiltration in the HOXC6 high expression group (**Figure 7H**, *P*<0.05). These results show that, compared with stage IV patients, the expression of HOXC6 had a greater impact on the tumor immune microenvironment in nonmetastatic CRC patients.

To evaluate the predictive value of HOXC6 expression on ICB response, the Tumor Immune Dysfunction and Exclusion (TIDE) algorithm was used in this analysis. TIDE uses a set of gene expression markers to evaluate two different tumor immune escape mechanisms, including the dysfunction of tumor-infiltrating cytotoxic T lymphocytes (CTLs) and the exclusion of CTLs by immunosuppressive factors. In nonmetastatic CRC patients, the HOXC6 high expression group had a significantly worse ICB response rate than the low expression group (**Figure 7I**, *P*<0.001). However, in mCRC patients, there was no significant difference between the HOXC6 high and low expression groups (**Figure 7J**).

The above results indicated that there was no significant difference in the prognosis of HOXC6 overexpression in patients with stage I- III disease. This may be caused by a higher proportion of infiltration of killer cells such as CD8+ T cells and a higher immunogenicity due to high HOXC6 expression. In stage IV patients, the overexpression of HOXC6 had a significantly worse prognosis, which may be related to a higher proportion of CD4+ T cell infiltration that promoted tumors, no difference in the infiltrated ratio of CD8+ T cells that killed tumors and a lower immunogenicity.

For response prediction of ICBs, the expression level of HOXC6 did not predict immunotherapy efficacy in mCRC patients. However, the results suggested that high expression of HOXC6 may be used as a potential biomarker for predicting immunotherapy efficacy, which may be used in nonmetastatic CRC treatment in the future. In other words, when immunotherapy is applied to stage II and III patients as adjuvant therapy such as ATOMIC (NCT 02912559) and POLEM trial (NCT 02912559), patients with high expression of HOXC6 may have worse immunotherapy efficacy.

## Discussion

In this study, we identified seven differentially expressed genes with prognostic significance in CRC compared to normal colon tissues. HOXC6, which had the greatest impact on the prognosis among these DEGs, was identified through cox univariate and multivariate prognostic analysis. Furthermore, high expression of HOXC6 was found to be significantly related to the tumor inflammatory microenvironment. Specifically, the expression of HOXC6 was significantly positively correlated with the infiltration ratio of immune cells, immune checkpoint marker expression, TMB and MSI score. Finally, in the stratified analysis according to clinical stages, we found that there was no significant difference in the expression of HOXC6 in different stages. In terms of prognosis analysis, the differential expression of HOXC6 in nonmetastatic CRC had no significant prognostic value, but in metastatic CRC, the high expression of HOXC6 was significantly correlated with worse prognosis. These results indicate that it is very worthwhile to explore the mechanism of TME orchestration caused by HOXC6 upregulation, which is expected to provide a candidate biomarker for CRC adjuvant immunotherapy cohort screening.

Although the prognostic value of HOX family genes has already been explored in bladder cancer (26) and laryngeal squamous cell cancer (27), this was the first systemic prognostic study in CRC. More importantly, it was found for the first time that in CRC, the high expression of HOXC6 was significantly related to the remodeling of the TME, including chemokine expression level, immune cell infiltration ratio, immune checkpoint expression level, TMB score, and MSI-H status. In CRC, HOXA13, HOXD13 and HOXC6 were reported to promote cancer progression, and HOXB13 was reported to suppress tumors. To the best of our knowledge, in CRC, only two studies have reported that high expression of HOXC6 could promote tumor metastasis by activating the classical WNT pathway and promote proliferation through the TGF-β/smad pathways (13, 25). In addition, HOXB13 was reported to inhibit the proliferation of right-sided colon cancer through the DNMT3B-HOXB13-C-myc regulatory axis (14).

In this study, high expression of HOXC6 was significantly positively correlated with the infiltration ratio of macrophages and neutrophils and the upregulation of immune checkpoint markers. Tumor associated macrophages (TAM) can be divided into M1 and M2 types, of which M2 has a tumor-promoting effect (28, 29). Monoclonal antibody drugs targeted three famous “don’t eat me” pathways on M2 macrophages to suppress the tumor promotion function of M2 macrophages (30–32). This strategy may serve as antitumor therapy in HOXC6 high-expression patients through more solid fundamental and animal experiments in the future. As the tumor progresses, locally infiltrated M1 macrophages gradually transform to M2 macrophages (33). Locally infiltrated neutrophils can also be divided into N1 and N2 types. Among them, the N2 type promotes tumors and NETs released after neutrophils apoptosis capture tumor cells and lead to their colonization and metastasis (34). The above various unfavorable factors cause the immunosuppressive microenvironment to promote tumor development and ultimately lead to tumor progression and poor prognosis.

Moreover, high expression of HOXC6 was also significantly positively correlated with the CD8+ T cell infiltration ratio, MSI-H score, TMB and dendritic cell infiltration ratio in our study. Although the increased infiltration of CD8+ T cells has the effect of killing tumor cells and is associated with a better prognosis, unfortunately tumors can induce the depletion or exhaustion of locally infiltrated CD8+ T cells by increasing the ICI on their surface, which leads to irreversible functional loss (35). High tumor load also has a reversible inhibitory effect on circulating CD8+ T cells, and immune function in the circulation can be reversed when the tumor load is reduced (36). Studies have found that MSI-H status can be used as an indicator of better prognosis in early-stage CRC, but in advanced patients, MSI-H had no survival benefit over MSS (37). The use of TMB as a predictive biomarker for immunotherapy is still controversial, and further research is needed (38). Locally infiltrated dendritic cells play an important role in the activation of CD8+ T cells *via* antigen presentation (39). High CD8+ T cells infiltration, MSI-H status, high TMB, and more dendritic cell infiltration were all favorable factors for antitumor ability. Inconsistent with this conclusion, the HOXC6 high expression group with these favorable prognostic factors did not have better survival in nonmetastatic CRC patients. High infiltration of M2 macrophages (40), elevated immune checkpoint markers (41) and high infiltration of CD4+ Tregs (42) resulted in CD8+ T functional loss. In summary, the combined results of various factors in the HOXC6 high expression groups did not result in a poor prognosis in nonmetastatic CRC.

For CD4+ T cells, the overexpression of HOXC6 may cause a higher proportion of CD4+ T cell infiltration, especially higher T cell follicular helper infiltration which was associated with favorable prognosis in mCRC patients (43). However, it has been reported that a high proportion of CD4+ T cell infiltration suggests a better prognosis in patients with CRC (44, 45). Tregs differentiated from naive CD4+ T cells could suppress the function of CD8+T cells (42). Moreover, CD8 + T cells stimulating capacity of CD4+ T cell follicular helper with increasing PD-1 expression in mCRC patients was inhibited by PD-1/PD-L1 pathway (43). These results combined with intratumoral metastasis promoting mechanisms induced by HOXC6 high expression (25) finally led to poor prognosis in mCRC patients.

There are some limitations in this study. Animal experiments should be performed to confirm our conclusion. More basic studies are needed to explain the lack of correlation between HOXC6 and TMB in mCRC. Further experiments are needed to confirm that the expression of HOXC6 may predict the efficacy of immunotherapy in nonmetastatic CRC. In addition, this is a preliminary study with practical limitation at this moment. Expression of HOXC6 should be quantified as absolute value further for clinical practice, such as the TPS, and IPS scores of PD-1 using IHC.

This study revealed that HOXC6 may serve as a potential biomarker for predicting the efficacy of immunotherapy in nonmetastatic CRC, which also provides clues for subsequent mechanistic studies.

## Data Availability Statement

The datasets presented in this study can be found in online repositories. The names of the repository/repositories and accession number(s) can be found in the article/**Supplementary Material**.

## Ethics Statement

Ethical review and approval was not required for the study on human participants in accordance with the local legislation and institutional requirements. Written informed consent for participation was not required for this study in accordance with the national legislation and the institutional requirements.

## Author Contributions

LQ, WH, SZ, and YY designed the study. LQ performed TCGA analysis. CY and DZ performed GEO analysis. WH performed statistical analysis. LQ, CY, and DZ wrote the manuscript. RB reviewed and polished the manuscript. All authors contributed to the article and approved the submitted version.

## Funding

This work was supported by National Natural Science Foundation of China (grant number: 81802883), Fundamental Research Funds for the Central Universities (grant number: 2018FZA7012) to WH, Zhejiang Basic Public Welfare Research Project (LGF18H160040) to RB, and National Natural Science Foundation of China (grant number: 82103102) to CY.

## Conflict of Interest

The authors declare that the research was conducted in the absence of any commercial or financial relationships that could be construed as a potential conflict of interest.

## Publisher’s Note

All claims expressed in this article are solely those of the authors and do not necessarily represent those of their affiliated organizations, or those of the publisher, the editors and the reviewers. Any product that may be evaluated in this article, or claim that may be made by its manufacturer, is not guaranteed or endorsed by the publisher.

## Supplementary Material

Supplementary Figure 1 Significantly upregulated HOX family genes in CRC tissues compared to normal colon tissues, however the upregulation had no effect on OS based on TCGA samples in UALCAN database.

Supplementary Figure 2 Significantly downregulated HOX family genes in CRC tissues compared to normal colon tissues, however the downregulation had no effect on OS based on TCGA samples in UALCAN database.

Supplementary Figure 3
**(A)** Mutation type analysis in HOXC6 high-expression group. **(B)** Mutation type analysis in HOXC6 low-expression group. **(C)** Top 10 significantly mutated genes in HOXC6 high-expression group compared to HOXC6 low-expression group (*P*<0.01). Ti: Transition; Tv: Transversion.
