# Supplementary figures and images for: The Effects of Differentially-Expressed Homeobox Family Genes on the Prognosis and HOXC6 on Immune Microenvironment Orchestration in Colorectal Cancer
Source: Front Immunol. 2021 Dec 7;12:781221. doi: 10.3389/fimmu.2021.781221 (PMC8688249; doi:10.3389/fimmu.2021.781221)

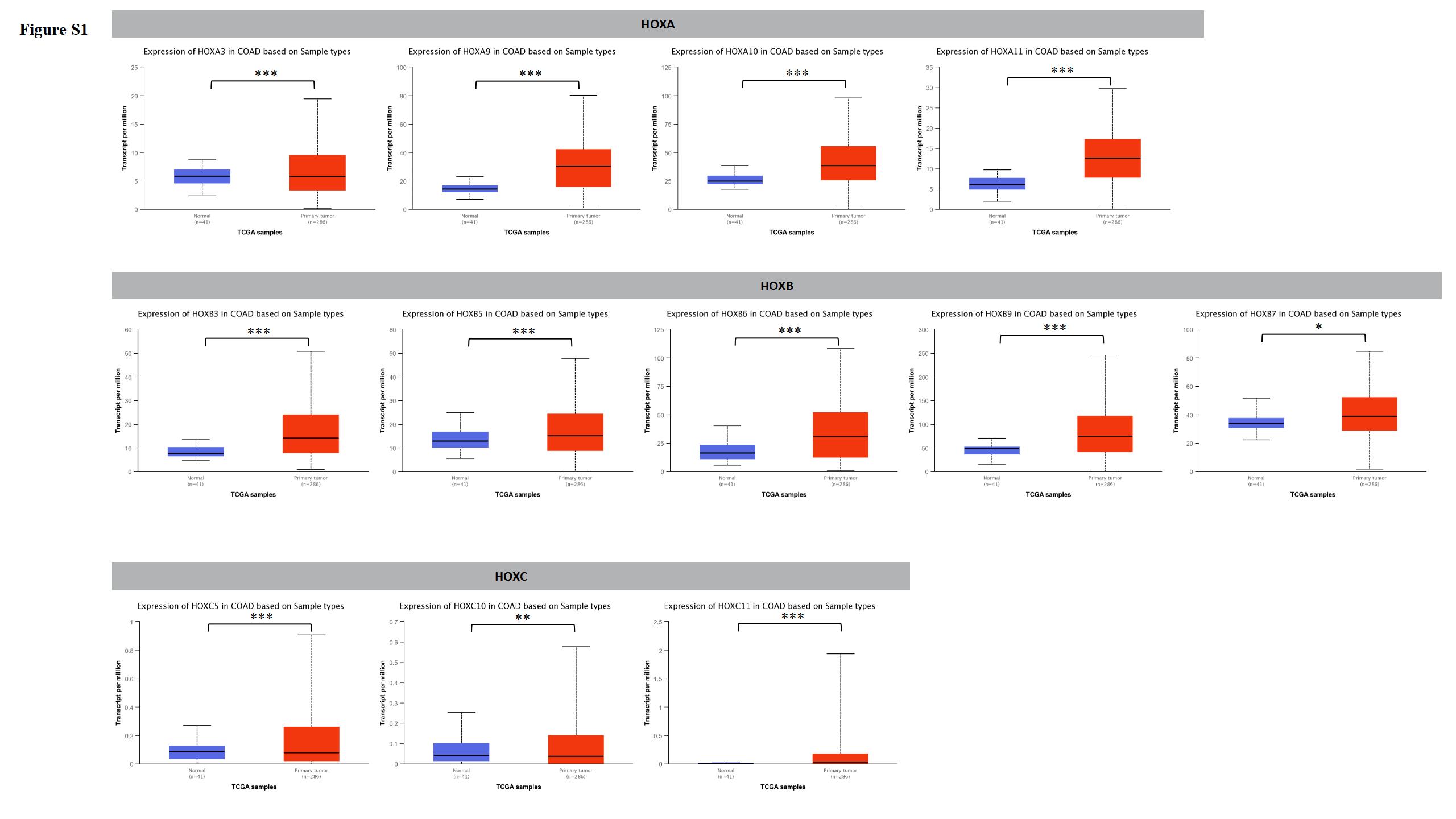

Supplement: Supplementary Figure 1 — Significantly upregulated HOX family genes in CRC tissues compared to normal colon tissues, however the upregulation had no effect on OS based on TCGA samples in UALCAN database. [file Image_1.jpeg]

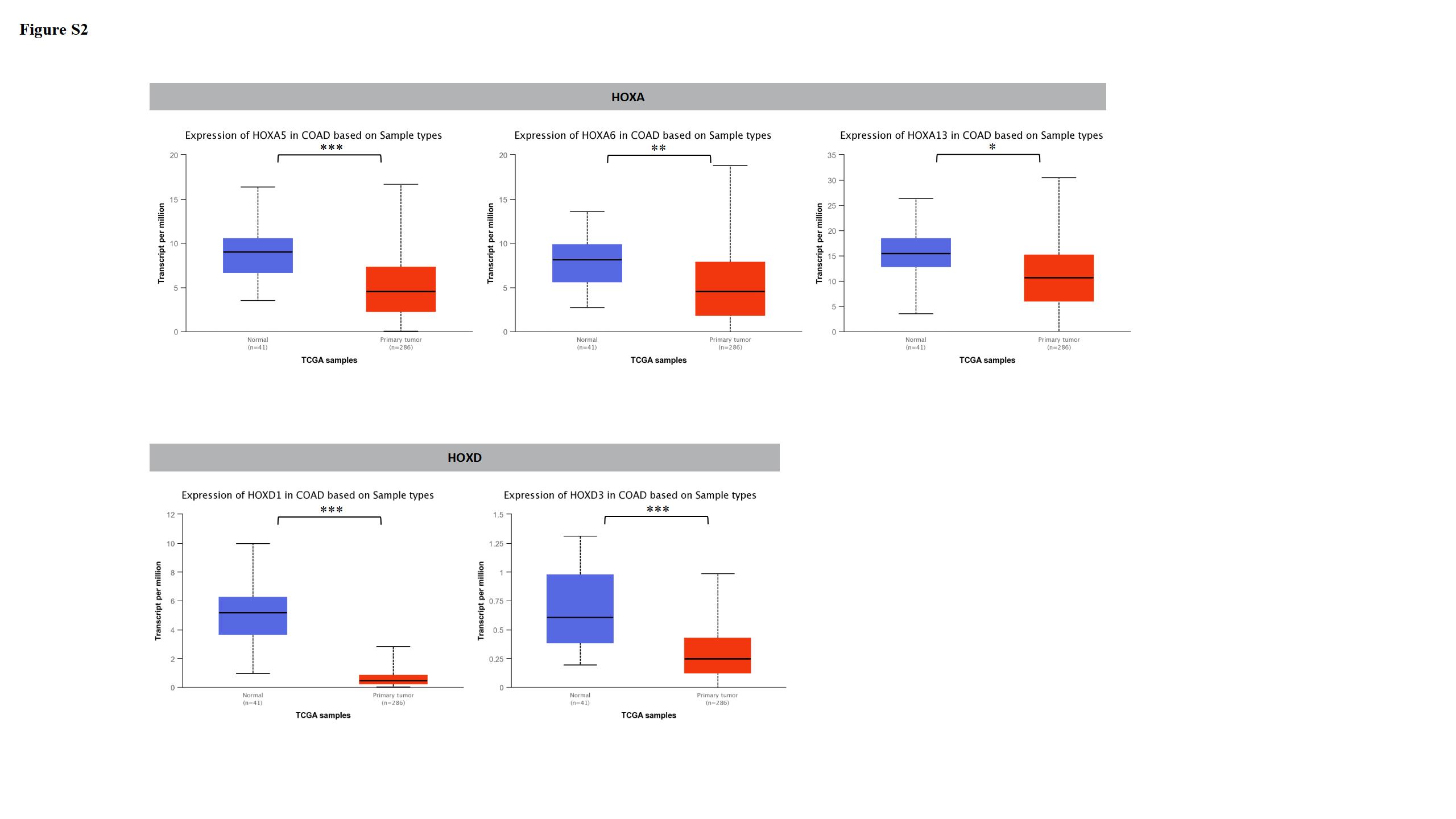

Supplement: Supplementary Figure 2 — Significantly downregulated HOX family genes in CRC tissues compared to normal colon tissues, however the downregulation had no effect on OS based on TCGA samples in UALCAN database. [file Image_2.jpeg]

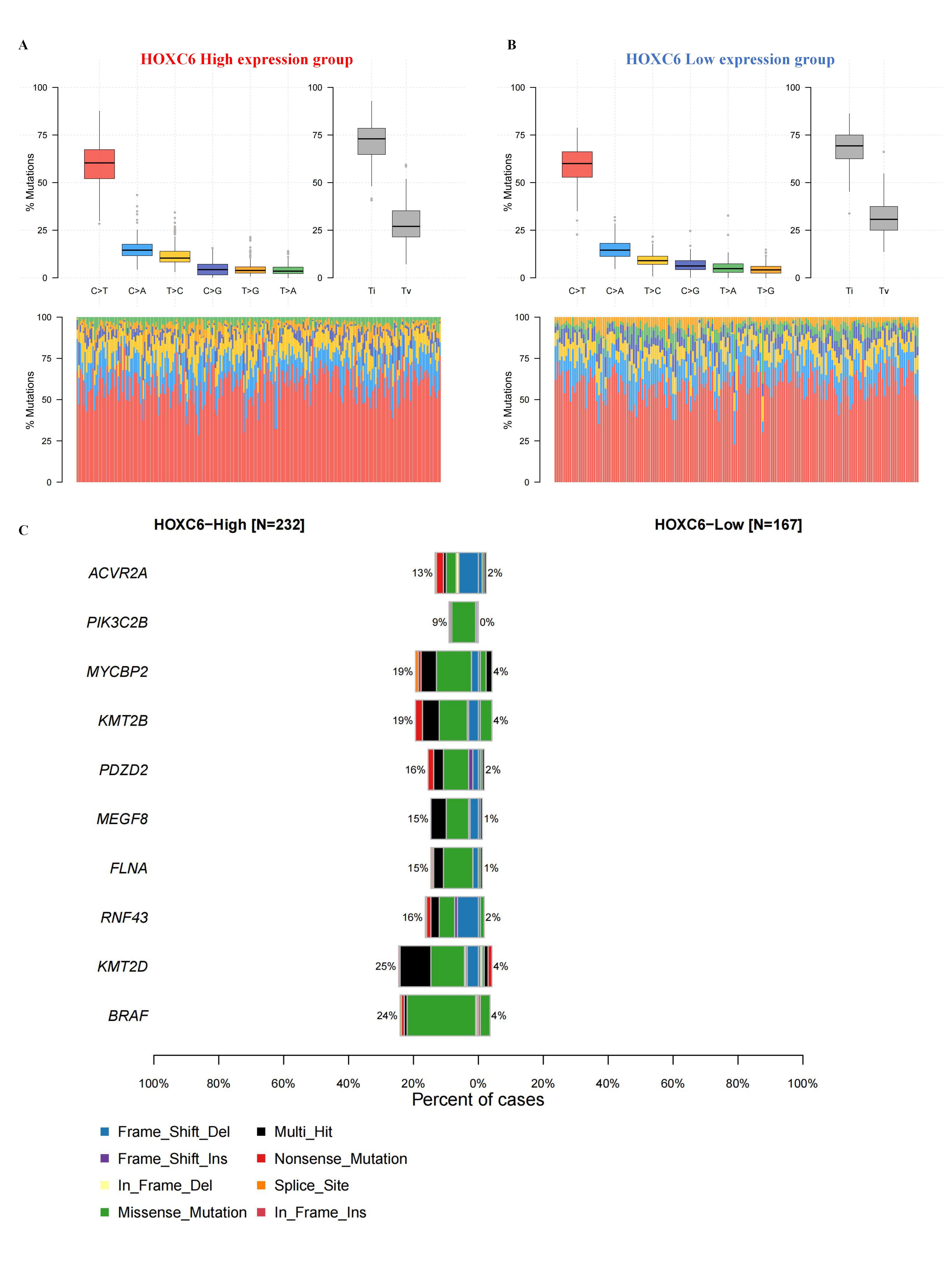

Supplement: Supplementary Figure 3 — (A) Mutation type analysis in HOXC6 high-expression group. (B) Mutation type analysis in HOXC6 low-expression group. (C) Top 10 significantly mutated genes in HOXC6 high-expression group compared to HOXC6 low-expression group (P<0.01). Ti: Transition; Tv: Transversion. [file Image_3.jpeg]
